# The effect of the leisure activities based on chess and cards for improving cognition of older adults: study protocol for a cluster randomized controlled trial

**DOI:** 10.1186/s13063-023-07528-1

**Published:** 2023-07-29

**Authors:** Xiaojuan Shi, Yanrong wang, Yueping Wu, Jiangping Li

**Affiliations:** 1 Department of Epidemiology and Health Statistics, School of Public Health, Ningxia Medical University, Yinchuan, 750004 China; 2 Key Laboratory of Environmental Factors and Chronic Disease Control, Ningxia Medical University, Hui Autonomous Region, Yinchuan, 750004 Ningxia China

**Keywords:** Leisure activities, Cognitive function, Promotion campaigns, Chess and cards

## Abstract

**Background:**

With the increase in age, the probability of cognitive impairment in the older people is increasing. More and more evidence shows that participating in leisure activities, especially chess and cards, is beneficial to the cognition and mental state of the older people. But the evidence for causal inference is limited. There is a need to conduct a fully powered randomized controlled trial (RCT) and long-term follow-up to test the effectiveness of intervention measures in improving cognitive function and mental state. This paper describes the methodology of a cluster RCT designed to determine the effect of chess and cards leisure activities on the cognitive function of the older people over 60 years old.

**Methods/design:**

A cluster RCT consisting of 8 clusters will be conducted in four regions of Ningxia, China (Helan, Litong, Qingtongxia, and Shapotou). Clusters will be randomly assigned to the advocacy intervention group, which is also delivered with free leisure activities tools (chess and cards), or the control group. A baseline survey will be conducted before the intervention. A mid-term and final survey will be carried out twelve and twenty-four months after the intervention, respectively. The primary outcome is a change in cognitive function, and the secondary outcomes are changes in anxiety, depression, and loneliness.

**Discussion:**

The results of this RCT will be helpful to (1) confirm the effectiveness of chess and cards leisure activities in improving the cognitive function of the older people over 60 years old; (2) determine the relationship between the frequency and duration of chess and cards leisure activities and cognitive function; (3) provide evidence of promoting participation in leisure activities through education campaigns and free provision of chess and cards tools; and (4) provide valuable information for successful aging.

**Trial registration:**

Chinese Clinical Trial Registry: ChiCTR2200066817. Registered on 19 December 2022.

**Supplementary Information:**

The online version contains supplementary material available at 10.1186/s13063-023-07528-1.

## Background

Driven by increased life expectancy and declining fertility rates, we are moving towards an aging world. Aging is not only the aging of the body and the degeneration of physiological functions, but also the decline of psychological and cognitive functions [1]. In the absence of effective treatment, the global financial and social burden caused by cognitive decline is set to rise [2].

According to severity, the cognitive decline of the older people can be divided into normal cognitive decline, subjective cognitive impairment, mild cognitive impairment (MCI), and dementia with age [3]. Dementia is the most severe level of impairment, which relieves symptoms but not cures [4, 5]. Interestingly, compared with dementia, the course of MCI is not always linear [6], and its outcome includes returning to normal aging, stabilizing, or progressing to dementia [7–10]. Thus MCI was considered the most effective period for dementia prevention and intervention [11–13].

To date, there are many clinical trials on the pharmacological and non-pharmacological treatment of MCI, but there is still no clear consensus on its treatment. The latter has the advantages of low cost, convenient implementation, and fewer adverse reactions. It has become the primary treatment recommended by clinics and a research hotspot in this field.

Leisure activities are defined as the voluntary use of free time for activities outside of daily life, which is one of the key components of a healthy lifestyle. After retirement, leisure time occupies most of the daily life of the older people. In recent decades, more and more attention has been paid to the role of leisure activities as a protective factor against cognitive function in the older people. A recent study shows that participating in leisure activities, not only as a prevention strategy, but even as an intervention in later life, may promote better cognitive performance through compensation or neural efficiency mechanisms [14]. The potential mechanism for the protective effect of leisure activities on cognitive function is still unclear. Most studies use the cognitive reserve hypothesis to explain the beneficial effects of leisure activities on cognitive function in old age. Increasing the function or plasticity of related neural networks may delay the onset of dementia or reduce cognitive decline, according to the cognitive reserve hypothesis [15].

A systematic review and meta-analysis [16] revealed that the active participation of the older people in leisure and recreational activities, especially intellectual activities, such as reading, playing chess and cards, can improve cognitive reserve, protect and restore cognitive function, and help reduce the risk of dementia in the older people. In addition, some studies have also shown that stimulating intelligence more frequently (such as cards, chess, mahjong, and crossword puzzles) was related to reducing the risk of dementia [17–19] and there may be a dose–response relationship. The reason why these stimulating intellectual games can reduce the risk of dementia may be that playing cards and chess, which involves attention, reasoning, memory, and initiative in cognitive fields, is a powerful and comprehensive stimulating activity for the brain [20, 21].

Meanwhile, playing chess and cards is group entertainment, which not only plays a positive role in cognitive function, but also promotes communication and provides significant social support and emotional comfort for the older people [22, 23]. Studies have shown that cards-playing activities can promote the mental health of middle-aged and older people by providing social communication and interaction [24].

Although previous studies have provided a great deal of epidemiological reference information about the association of leisure activities with cognitive function, the epidemiological evidence linking specific leisure activities with reducing cognitive decline is not enough to indicate whether these activities have any direct preventive or delaying effects. So far, the evidence from randomized controlled trials supporting the cognitive benefits of leisure activities in the older people through chess and cards is still limited. Most research designs are cross-sectional studies [25] or longitudinal studies [26], and causal inferences cannot be made. Individual randomized controlled trials with a small sample size on cognitive leisure activities are limited to cognitive impairment samples [27, 28]. Therefore, intervention research is needed to prove the causal relationship between chess and cards leisure activities and cognitive function in the population, and to verify whether chess and cards leisure activities are an effective non-drug treatment for the older people. In recent years, social media have been a source of information about cognitive impairment prevention and treatment. This is because massive promotion campaign is also one of the most important aspects of health work. We expect the elderly to voluntarily give up problematic living habits and adopt active lifestyles to delay the decline of cognitive function after being advertised.

Furthermore, there is no single, large-scale, and robust design study to measure the cognitive intervention effect of free chess and cards leisure activities on the older people. To solve this problem, we designed a more convenient and economical cluster randomized controlled trial to avoid contamination and evaluate the real effect of the intervention. We aim to maximize the potential health benefits of chess and cards activities for the older people through intervention. We will not only pay attention to communication campaigns, but also the availability of tools (chess and cards), and improving the activities and atmosphere.

We hypothesize that compared with the older people in the control group without any intervention, the long-term collective leisure activities of chess and cards for the aging population can not only promote the older people to maintain a good daily emotional state, but also effectively avoid or prevent the cognitive decline of the older people. In addition, this kind of activity is simple and popular among the older population, and is easy to be implemented among the older people. If the hypothesis is true, it means that preventive intervention for the alterable lifestyle of the older people can improve the clinical manifestations of cognitive impairment in the older people and delay the onset of dementia syndrome, thus contributing to the healthy aging of the brain, which has great public health significance.

### Objectives

This study determined the long-term effectiveness of chess and cards leisure activities in improving cognitive and psychological status in older adults compared with those who did not pursue these activities.

#### Primary objective

To investigate the effects of leisure activities using chess and cards entertainment tools on objectively measured cognitive function compared to usual practice at 24-month follow-up.

#### Secondary objectives

To investigate the impact of intervention over the medium (assessed at 12 months) and long term (assessed at 24 months) on:

- Cognitive function
- Psychosocial variables (anxiety, depression, and loneliness)
- Frequency and duration of chess and cards activities
- Daily activity function
- Knowledge of cognitive impairment prevention

### Trial design

Two years will be spent on this cluster-randomized controlled study. Participants in this study will be older people over 60 years old. It is hypothesized that chess and cards leisure activities can delay cognitive decline, improve neuropsychiatric symptoms of depression, anxiety, and loneliness, and enhance health.

## Methods: participants, interventions, and outcomes

### Study setting

**Fig. 1:**
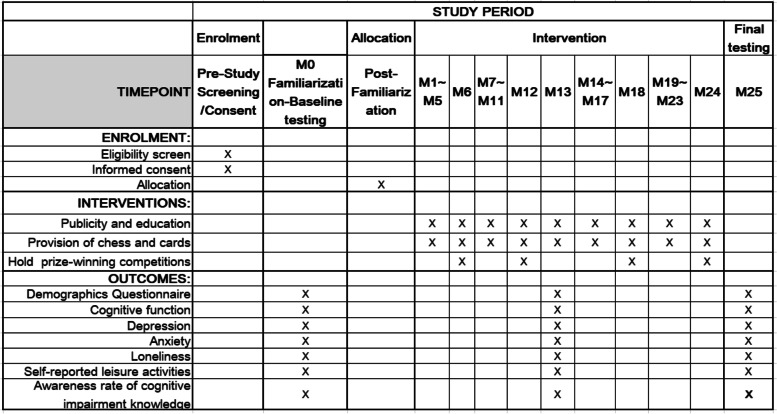
Schedule of enrollment, interventions, and assessments for this study

**Fig. 2:**
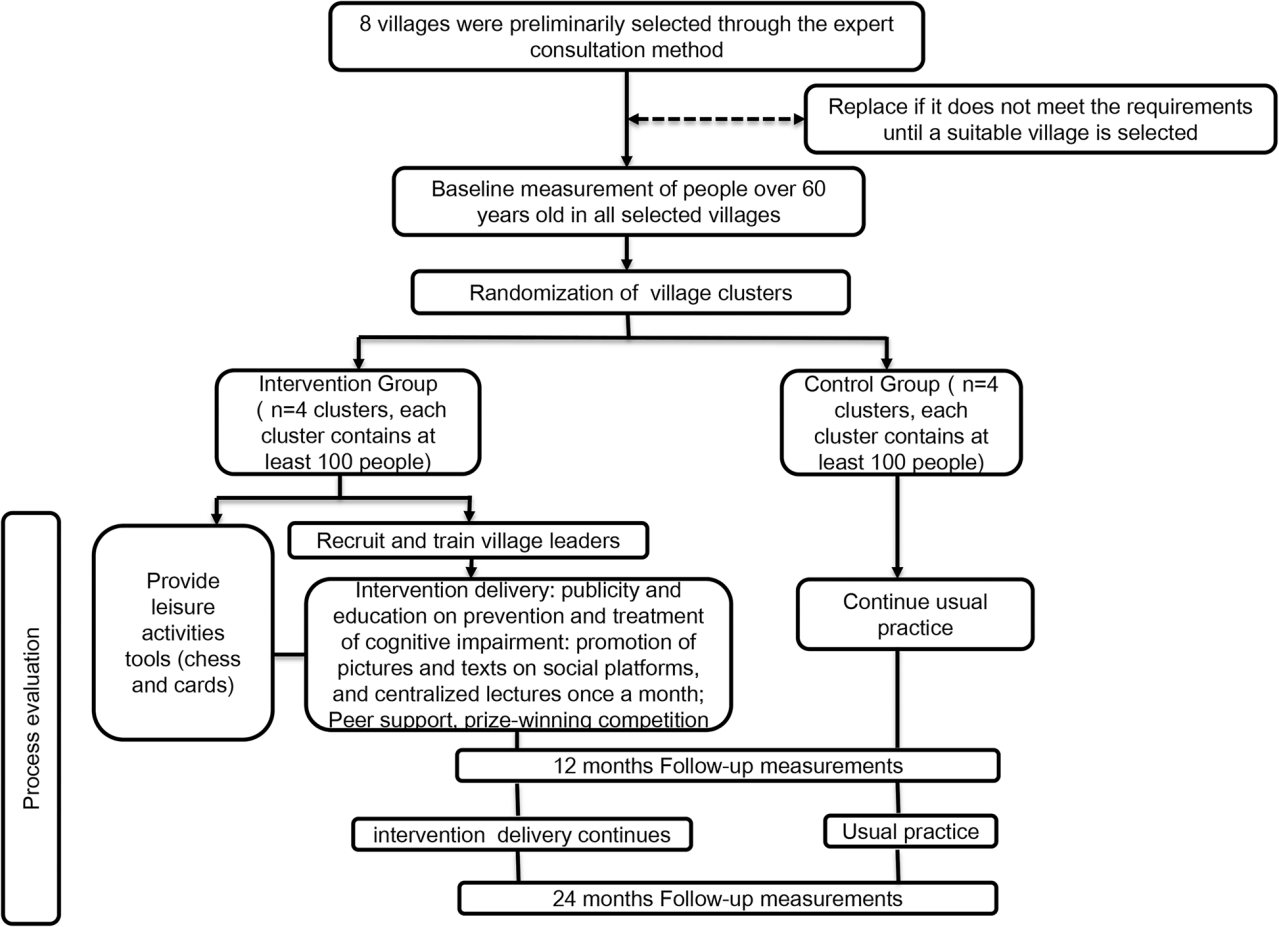
Flow chart of the study design

This study is a two-arm, open-label randomized trial and will be conducted in the Ningxia Hui Autonomous Region of China. The recruitment of observation objects is expected to be completed in June 2023. This study protocol follows the standard protocol for clinical trials in accordance with the SPIRIT 2013 statement [29] and follows the CONSORT statement for clinical trial transparency [30]. A flow diagram is provided in Fig. 1 which clearly outlines the study’s recruitment process. The study is registered on the Chinese Registry of Clinical Trials platform under the license ChiCTR2200066817. Research design and consent have been approved by the Ethics Committee of Ningxia Medical University (No.2022–025). Figure 2 shows the design flow chart for this study.

### Cluster and participant recruitment

The study areas are selected by an expert consultation method. First, we selected Helan County of Yinchuan, Litong District of Wuzhong City, Qingtongxia City, and Shapotou District of Zhongwei with high-quality public health work to carry out this study. In the second stage of sampling, taking villages as the basic unit, two villages are randomly selected from the eligible villages in the above four project research areas, and the selected villages are matched (1:1) as control villages and intervention villages respectively. The matching principle is based on the county to which the village belongs.

The selected villages need to meet the following conditions:

- The population aged 60 is ≥ 100.
- Residents have fewer daily leisure activities
- Village cadres/village doctors are serious and responsible.

### Inclusion and exclusion criteria

#### Inclusion criteria

Participants who meet the following criteria will be eligible:

- Resident residents who have lived in the local area for more than 6 months.
- Age ≥ 60 years old.
- Volunteer to participate in the research after fully understanding the project information.

#### Exclusion criteria

The participant may not enter the study if any of the following apply:

- In the study period, those who did not live in the village due to hospitalizations, traveling, or other reasons did not participate in the study.
- Aphasia, deafness, blindness, paraplegia, or other physical diseases and/or inability to communicate normally with others.
- Suffering from major diseases (severe heart, liver, kidney, lung, and metabolic diseases), nervous system diseases that can cause brain dysfunction, or those who have been at the end of their lives.
- Patients with severe mental disorders, depression, and dementia who have been diagnosed.
- Loss of daily living abilities.

Participants will receive concise information explanatory statements and an oral description of research details once they agree to participate. An explanation of the study's purpose, the procedure, the use of participant data, requirements of study participants during the 2-year activity, potential benefits and risks, how their information will be kept confidential, and contact information for any questions related to the project will be provided to participants both orally and in writing. Before any investigation is conducted, written consent will be obtained. At any time, participants will be informed that they can withdraw from the study and withdraw their consent. During the study, participants will be asked to maintain daily physical activity and eating habits. There will be no biological specimens collected in the present study.

As a reward for participating in the survey, participants will receive 20 RMB worth of paper towels and detergent. In medicine, the way to ensure participation in medical research through incentives is quite sensitive and controversial [31]. In the present study, although the compensation for the participants seems to be extremely low, the research conforms to the usual ethical standards of human subject research and will not bring additional risks to the subjects. We do not attract and supervise participants through compensation, they all participate in the research voluntarily.

### Interventions

#### Intervention goal

In the 24 months after the intervention, the older people over 60 years old actively participated in chess and cards leisure activities, improving cognitive function and neuropsychiatric symptoms as a result.

### Main intervention components

#### A KAP mode-based approach to intervention

The Knowledge-Attitudes-Belief-Practice (KABP) Model is an important part of the theory of healthy behavior change [32]. The theory holds that acquiring knowledge leads to changes in beliefs and the ultimate goal of establishing healthy behaviors. Positive, correct beliefs and attitudes are built upon knowledge and information. Beliefs and attitudes are the drivers of healthy behavior change. Consequently, this theory plays a significant role in changing unhealthy behaviors and cultivating beneficial behaviors [33]. The purpose of intervention is to improve the knowledge of cognitive impairment, change attitudes, actively participate in chess and cards leisure activities, and then improve cognitive function. Therefore, we adopt strategies to advertise the study, improving the participants' engagement and disseminating the findings.

### Intervention group

Each participant in the intervention group will be given personalized chess and cards leisure activities tools (chess, military chess, gobang, checkers, poker, long cards, etc.) according to their preferences. In addition, we provide education campaigns on the prevention and treatment of cognitive impairment for all residents in the village. Promotion is achieved by publishing relevant popular science content on the village's social platform. Every month, the fixed host convenes the whole village over 60 years old to give a lecture on cognitive impairment prevention. Education focuses on leisure activities and their benefits. Participants are encouraged to plan their leisure time reasonably and take the initiative to participate in various leisure activities, especially social leisure activities such as playing cards and chess. The recommended frequency of chess and cards activities is 5–6 times a week, for at least 1 h. Each village will appoint a responsible person and receive training to motivate their neighbors to actively participate in chess and cards leisure activities. Social support from neighbors and family members will be encouraged through participation in regular activities inside and outside of life. The intervention time was 2 years.

### Control group

Villages assigned to the usual practice control arm were not given any additional activities and were instructed to continue with their normal, everyday activities and hobbies throughout follow-up. Participants in the control group will make the same research measurements at the same time as those in the intervention group.

### Relevant concomitant care permitted or prohibited during the trial

Implementing the intervention will not require alteration to usual care pathways (including the use of any medication) and these will continue for both trial arms. There are no restrictions regarding concomitant care during the trial.

### Outcomes

Uniformly trained researchers will evaluate results at three time points: baseline, 12 months, and 24 months following baseline. During the baseline survey, researchers will fully explain the purpose and content of the study to the participants. If participants are willing to join the study, they will sign a written informed consent. Measurements will only be made after informed consent is given. Cognitive function, depression, anxiety and loneliness, frequency of leisure activities, demographics, and awareness rate of cognitive impairment will be measured. The schedule of enrolment, interventions, and assessments is outlined in Fig. 1.

### Primary outcomes

#### Cognitive function

The Mini-Mental State Examination (MMSE) [34, 35] and Montreal Cognitive Assessment (MoCA) [36] are simple, low cost and noninvasive methods of screening MCI due to Alzheimer’s disease (AD). Numerous studies stated that MMSE and MoCA accuracy in detecting MCI due to AD was inconsistent when selecting different reference standards as the “gold standard. In addition, they selected different cutoff values for positivity. To date, there is still no consensus on which neuropsychological test performs better at detecting MCI due to AD. In addition, international criteria are more suitable as a reference standard for evaluating diagnostic tools' accuracy. Studies have shown that the combined use of MMSE and MoCA scales for cognitive impairment diagnosis is more accurate than MMSE or MoCA scales alone. Therefore, we use MMSE and MoCA together to assess cognitive function. The ability to perform activities of daily living (ADL) is a vital part of the assessment in neurologic patients. An ADL scale was used to evaluate subjects' functional status in real life. It consists of two parts: the Physical Self-Maintenance Scale (PSMS) and Instrumental Activities of Daily Living (IADL).

### Secondary outcomes

#### Depression, anxiety, and loneliness

Depression will be assessed through the Chinese version of the 30-item depression scale for the older people (GDS-30), a popular screening tool for depression that has been in use for more than 30 years [37]. A score of 11 or higher suggests depression. A norm-referenced scale, the Self-Rating Anxiety Scale (SAS), is widely used as a screening tool for anxiety disorders [38, 39]. Loneliness will be assessed using the UCLA Loneliness Scale, which has satisfactory reliability and validity [40].

#### Self-reported chess and cards leisure activities

Participants will be asked to complete a questionnaire on their participation in chess and cards leisure activities. They will also be asked to estimate the frequency and duration of their participation in chess and cards leisure activities. Information on sleep duration and quality, smoking status, drinking status, and exercise status will also be collected through self-reports.

#### Demographic

During their baseline visit, we obtained the following covariates from face-to-face interviews: age, sex, education level, living conditions (living alone or with others), family relations, and monthly income. In addition, participants will report previous illnesses (such as hypertension and diabetes) and a family history of dementia. At each follow-up, participants will be asked if there has been any change in these areas. If yes, it will be revised and the latest follow-up information will be used.

#### Awareness rate of cognitive impairment knowledge

By comparing the awareness rate of cognitive impairment knowledge before and after the intervention, the effect of education campaign will be evaluated. The contents will focus on basic cognitive impairment prevention knowledge.

### Sample size

PASS 15.0 software calculated the sample size. Power calculation indicated that with a sample size of 4 clusters in each group and 100 observed objects in each cluster, this study would have 88% power to detect the change in cognitive function with a mean of 1, a standard deviation of 2, and a two-tailed significance level of 5%. As a result of the correlation coefficients being set to 0.050 within clusters, and the coefficients of variation being set to 0.650, four clusters in each group, resulting in a total of 800 people to be recruited. The power of RCT over 80% is considered acceptable [41], so the sample size calculated in this study will be eligible.

### Assignment of interventions: allocation

#### Sequence generation

In this cluster randomized controlled trial (RCT), 8 villages will be recruited. A village is a cluster. To reduce contamination risk, clusters will be randomized at the village level. Using a computer-generated list, statisticians at Ningxia Medical University randomly assigned villages (clusters) to one of the two groups and stratified them according to the counties to which villages belong before allocation. The randomization will be performed using a computer-generated randomization table in Microsoft Excel.

Randomization will be carried out after the cluster completes the baseline measurement. These clusters will be recruited from Helan County, Litong District, Qingtongxia City, and Shapotou District in Ningxia, China. Our goal is to recruit 800 over-60 s in 8 clusters. Clusters will be randomly assigned to accept one of the following conditions: (1) intervention groups that distribute leisure activities tools (chess and cards) free of charge and promote them, or (2) control groups (routine).

### Allocation concealment and blinding

Given the nature of the study, participants can't turn a blind eye to the interventions they receive. The researchers collecting data and managing participants are blinded to the groups and will not know the groups until the data analysis is complete.

### Data collection and management

Research assistants blinded to the allocation will be trained in data collection. Participants will be observed and surveyed using questionnaires throughout the study period. The medium-term and long-term effects of the intervention were evaluated by repeated measurements using the same standardized procedures before, 12 months, and 24 months after the intervention.

All collected data will be kept strictly confidential and properly preserved, and all relevant regulations will be observed. Each participant is identified by a unique code instead of a name. All research data can only be obtained by designated research team members before analysis. These data were initially registered on paper questionnaires by investigators and then entered into electronic data through Epidata 3.1 software after the investigation. Electronic data is stored on password-protected computers, and paper questionnaires are stored in locked filing cabinets. When collecting data, the project manager will check all data for omissions or errors and record any problems with the data. Before analyzing the data, the lead researcher (PI) and statistician will review it again. Information collected from the study will be preserved until the project is completed. From the date when the study results are published, they will be preserved for at least 5 years.

### Statistical methods

#### Primary and secondary outcomes

The primary purpose of the analysis was to investigate the effects of multicomponent interventions (targeted promotion campaigns, providing free cards and chess leisure activities tools) on objectively measured cognitive function and neuropsychiatric state, compared with the usual practice at 24-month follow-up.

The primary outcome analysis is powered to detect a significant difference in cognitive function and neuropsychiatric status in the older people over 60 years at the end of a 24-month follow-up period. Linear multilevel models will be used to analyze the cognitive function and other neuropsychiatric indicators as outcome variables. Levels indicate the clustering of residents within villages. We take the randomly divided classification variables as independent variables (the intervention group will be compared with the control group), and age, family history of dementia, baseline value, smoking, drinking, and sleep quality as confounding factors. In these linear multilevel models, village clusters will be included as a random effect to simulate the heterogeneity of residents in villages. The model will use restricted maximum likelihood estimation.

After the survey, the differences in baseline characteristics between participants who completed the whole survey and those who dropped out of the study will be compared. Multiple interpolation and sensitivity analyses of missing data are carried out to evaluate the influence of missing data on the obtained results. The effect size of the protocol will be determined by the subjects who participated in the study and completed follow-up. Secondary outcomes measured at other time points will be analyzed using similar methods. In addition, we will use repeated measurement variance analysis to evaluate the changes in the main outcome indicators of each protocol at each time point. Subgroup analyses are planned for baseline characteristics that might affect the primary outcomes.

The p values of all tests are two-sided and the test level α is 0.05. Estimates will also be reported with a 95% confidence interval.

### Process evaluation

A combination of questionnaires, interviews, and field observations will be used to assess the process. The purpose of process assessment is to understand: participants' different experiences of intervention measures; any differences between expected and observed results; sustainability of intervention programs; possible contamination between the intervention group and the control group; and any unexpected events in the process that are not part of the results of the study.

Attendance at communication campaigns will be recorded. Each cluster will have a special person to check intervention measures implementation. All activity processes will be recorded. In case of important modifications, they will be explained to the experimental participants in accordance with relevant regulations and agreed, and the modifications will be made in the trial registration center at the same time. After each follow-up, the project researchers will analyze the data and report the results to the PI. During the experiment, when force majeure fails to continue intervention or other situations must be terminated, the PI will make the final decision to terminate the trial.

### Oversight and monitoring

#### Composition of the coordinating center and trial steering committee

Ningxia Medical University will serve as the coordination center. In view of the low risk associated with this study, no independent trial steering committee will be established for this study.

#### Composition of the data monitoring committee, its role, and reporting structure

The School of Public Health of Ningxia Medical University will independently monitor the data. They are responsible for verifying the qualifications of the participants, the written informed consent, compliance agreements, and the accuracy of the data collected. Only investigators and members of the data center will have access to anonymous data.

### Ancillary and post‑trial care

Although the team in this study aims to mitigate the risks associated with the intervention, minor harm may nonetheless occur. Compensation is not provided for participants who are injured in this research project. In case of injury, participants will be advised to contact Ningxia Medical University's Ethics Committee.

### Frequency and plans for auditing trial conduct

The Project Management Group meet to review trial conduct once a month. The independent Data Monitoring and Ethics Committee meet to review conduct throughout the trial period. An independent party will audit and report the results.

### Plans for communicating important protocol amendments to relevant parties

Any protocol modifications will be reviewed by Ningxia Medical University and then revised in the Chinese Clinical Trial Registry. All relevant information will be shared among researchers.

### Dissemination plans

The results of this study will be published in peer-reviewed journals.

### Patient and public involvement

In the design stage of the study, we gathered the older people over 60 years old to discuss which leisure activities are more appropriate, and discussed what kind of outcome indicators could be collected. This has a positive effect on the formulation of our protocol. In addition, during the study, researchers will visit the site regularly and adopt diversified methods to obtain the opinions of the research objects, improving the public’s and participants’ engagement throughout the study process and disseminating the findings.

## Discussion

With the increase in age, the probability of cognitive impairment in the older people is on the rise [42, 43]. More and more evidence shows that participating in cards and chess leisure activities is beneficial to the cognitive and psychiatric state of the older people [18, 19]. Although previous cross-sectional or cohort studies have shown beneficial prospects for leisure activities [44, 45], the quality of the study design is low. Many previous studies have not fully considered baseline cognitive state and other confounding factors, both of which may lead to bias, and no effective cluster randomized controlled trials are underway. Advantages of this study include robust randomized controlled trial design, randomization at the cluster level to reduce contamination, large population, including medium-term and long-term follow-up measures, and strict objective scale measurement as the main results. In addition, the uniqueness of this study is that it will clarify the influence of the frequency and duration of participation in chess and cards activities on cognitive function. Inevitable limitations of this study include: First, due to the nature of this study, participants cannot turn a blind eye to the interventions they receive, and if researchers interact with subjects, they cannot keep blind to researchers, which may lead to information bias. Second, basic information, the duration and frequency of leisure activities are reported by the subjects themselves, which may lead to recall bias. Nevertheless, the present study will fill gaps in the literature. This study will help to build an evidence base around the effectiveness of interventions to reduce cognitive impairment in the older people, and provide valuable information for successful aging.

### Trial status

The recruitment time of this study is from March to June 2023. The anticipated period is 24 months, and the study is expected to be completed in June 2025.

## Supplementary Information

**Additional file 1. **

## Data Availability

Data will be made available after the main trial analyses have been completed on reasonable request from researchers with ethics approval and a clear protocol. The informed consent form is in Chinese, and the relevant materials of informed consent are available, on request, from the corresponding author.

## Abbreviations

RCT: Randomized controlled trial
MCI: Mild cognitive impairment
CONSORT: Consolidation Standards of Reporting Trials
KABP: Knowledge-Attitudes-Belief-Practice
MMSE: Mini-Mental State Examination
MoCA: Montreal Cognitive Assessment
AD: Alzheimer’s disease
ADL: Activities of daily living
PSMS: Physical Self-Maintenance Scale
GDS: The Geriatric Depression Scale
SAS: Self-rating Anxiety Scale
